# Symbiont-Mediated Protection of *Acromyrmex* Leaf-Cutter Ants from the Entomopathogenic Fungus Metarhizium anisopliae

**DOI:** 10.1128/mBio.01885-21

**Published:** 2021-12-21

**Authors:** Gaspar Bruner-Montero, Matthew Wood, Heidi A. Horn, Erin Gemperline, Lingjun Li, Cameron R. Currie

**Affiliations:** a Department of Bacteriology, University of Wisconsin, Madison, Wisconsin, USA; b Department of Genetics, University of Cambridge, Cambridge, United Kingdom; c Department of Chemistry, University of Wisconsin, Madison, Wisconsin, USA; d School of Pharmacy, University of Wisconsin, Madison, Wisconsin, USA; e Department of Energy Great Lakes Bioenergy Research Center, University of Wisconsin, Madison, Wisconsin, USA; Harvard University

**Keywords:** defensive symbiosis, symbiont acquisition, fungus-growing ants, host-parasite interactions, antifungal

## Abstract

Many fungus-growing ants engage in a defensive symbiosis with antibiotic-producing ectosymbiotic bacteria in the genus *Pseudonocardia*, which help protect the ants’ fungal mutualist from a specialized mycoparasite, *Escovopsis*. Here, using germfree ant rearing and experimental pathogen infection treatments, we evaluate if *Acromyrmex* ants derive higher immunity to the entomopathogenic fungus Metarhizium anisopliae from their *Pseudonocardia* symbionts. We further examine the ecological dynamics and defensive capacities of *Pseudonocardia* against *M. anisopliae* across seven different *Acromyrmex* species by controlling *Pseudonocardia* acquisition using ant-nonnative *Pseudonocardia* switches, *in vitro* challenges, and *in situ* mass spectrometry imaging (MSI). We show that *Pseudonocardia* protects the ants against *M. anisopliae* across different *Acromyrmex* species and appears to afford higher protection than metapleural gland (MG) secretions. Although Acromyrmex echinatior ants with nonnative *Pseudonocardia* symbionts receive protection from *M. anisopliae* regardless of the strain acquired compared with *Pseudonocardia*-free conditions, we find significant variation in the degree of protection conferred by different *Pseudonocardia* strains. Additionally, when ants were reared in *Pseudonocardia*-free conditions, some species exhibit more susceptibility to *M. anisopliae* than others, indicating that some ant species depend more on defensive symbionts than others. *In vitro* challenge experiments indicate that *Pseudonocardia* reduces Metarhizium conidiospore germination area. Our chemometric analysis using matrix-assisted laser desorption/ionization mass spectrometry imaging (MALDI-MSI) reveals that *Pseudonocardia*-carrying ants produce more chemical signals than *Pseudonocardia*-free treatments, indicating that *Pseudonocardia* produces bioactive metabolites on the *Acromyrmex* cuticle. Our results indicate that *Pseudonocardia* can serve as a dual-purpose defensive symbiont, conferring increased immunity for both the obligate fungal mutualist and the ants themselves.

## INTRODUCTION

Microbial symbionts provide ecological and physiological services, from supplying nutrients to protection from pathogens. Symbiont-derived protection can be crucial for the health and fitness of some hosts, particularly in insects (1–8). While the primary models of symbiont-derived protection in insects involve intracellular and gut symbiotic bacteria (9–16), less is known about insect-associated ectosymbiotic bacteria and their effects on host defense (17–20). Some insect-associated ectosymbiotic bacteria produce antimicrobial compounds that protect their insect host, its offspring, or other symbiotic partners, such as in termites, southern pine beetles, beewolves, bees, and fungus-growing ants (21–28).

Leaf-cutter ants (*Acromyrmex* and *Atta* species) are the most derived species of the attine fungus-growing ants, are dominant herbivores in the Neotropics, and are important agricultural pests (29, 30). The ants cultivate a fungal garden by supplying it with fresh plant material, and in return, the fungus Leucoagaricus gongylophorus produces hyphal swellings on which the ants feed (29, 31, 32). The fungal garden is susceptible to infection by a specialized mycoparasite in the genus *Escovopsis* (33–35). To help protect their gardens against pathogens, the ants utilize both physical and chemical measures, such as grooming the garden (36) and using antibiotic-secreting metapleural glands (36–40). In the genus *Acromyrmex*, garden-tending workers carry an antibiotic-producing ectosymbiotic *Pseudonocardia* bacterium (*Actinobacteria*) on specialized structures on their exoskeleton that help to protect the fungus garden from *Escovopsi*s (36, 41, 42).

*Pseudonocardia* symbiont transmission occurs from caregiver nestmates to callow workers and occurs within a narrow window of time after worker eclosion (43, 44). This quick transmission likely helps ensure the specificity and fidelity of native symbionts and reduces the opportunity for colonization by other bacteria. As predicted by this specialized mode of transmission and narrow window of acquisition, studies report evidence for the presence of just a single strain of *Pseudonocardia* on individual ants and within colonies (43, 45, 46). Since the origin of the attine-*Pseudonocardia* symbiosis, the ants have acquired free-living *Pseudonocardia* strains multiple times, and over the evolutionary history of this association, switches between ant species and some *Pseudonocardia* lineages have occurred (22, 47).

In many species of *Acromyrmex*, nestmate workers harbor an abundance of *Pseudonocardia.* The coverage of the ectosymbiont increases exponentially from initial inoculation through 10 to 15 days posteclosion, resulting in the colonization of virtually the entire ant exoskeleton. *Pseudonocardia* abundance then decreases 25 days posteclosion (44). The heavy abundance of the *Pseudonocardia* symbiont likely facilitates the application of antimicrobial compounds for the protection of fungal gardens from *Escovopsis*. Additionally, given that many entomopathogens infect their host by penetrating the exoskeleton, it has been suggested that *Pseudonocardia* coverage may prevent spore germination of fungal pathogens on workers by acting as a physical barrier on the ant exoskeleton (33, 41). Mattoso and colleagues (82) showed, using a single colony of Acromyrmex subterraneus subterraneus, that removing *Pseudonocardia* from the exoskeleton of workers increases their susceptibility to the entomopathogenic fungus Metarhizium anisopliae, a common insect pathogen. Although much is known about the ability of *Pseudonocardia* to inhibit *Escovopsis*, our understanding of the protective role of *Pseudonocardia* against Metarhizium across *Acromyrmex* ants is limited.

Here, we explore the role of *Pseudonocardia* in the *Acromyrmex* leaf-cutter ants as a protective partner against the entomopathogenic fungus Metarhizium. Specifically, we evaluate whether (i) *Pseudonocardia*-derived protection against Metarhizium is found broadly across *Acromyrmex* ant species, (ii) there is a trade-off between *Pseudonocardia* and metapleural gland (MG) secretions on ant susceptibility to the pathogen, (iii) the acquisition of nonnative *Pseudonocardia* affects individual ant survival, (iv) the abundance of nonnative and native *Pseudonocardia* increases protection, and (v) *Pseudonocardia* protects the ants by the production of bioactive compounds *in vitro* and *in situ*.

## RESULTS

### *Pseudonocardia*-mediated protection from Metarhizium.

**FIG 1:**
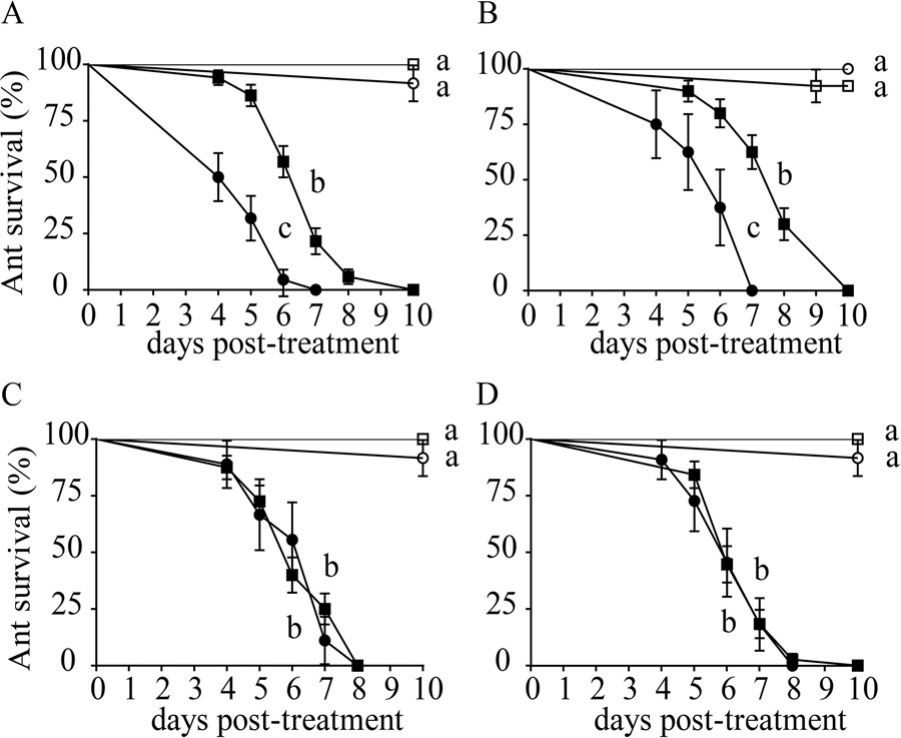
*Pseudonocardia* as a defensive symbiont in four species of *Acromyrmex* leaf-cutter ants. Survivorship curves of *Acromyrmex* workers with *A. echinatior* (A), *A. octospinosus* (B), *A. laticeps* (C), and A. niger (D) carrying *Pseudonocardia* from their own colony (native) (■ and □) or under a *Pseudonocardia*-free condition (● and ○), exposed to Metarhizium (solid symbols) or a control solution (open symbols) of sterile deionized water + 0.01% Tween 20. Error bars represent standard error. Letters represent significant differences from one treatment to another at a *P* value of <0.05 in pairwise comparisons using a Kaplan-Meier pairwise test.

Ant survival was significantly greater for control-treated ants than for those exposed to Metarhizium (Wald = 32.45, df = 1, *P < *0.0001). Overall, ants carrying their own *Pseudonocardia* (native) survived 2.24 times more than *Pseudonocardia*-free ants (Wald = 59.67, df = 1, *P < *0.0001) (Fig. 1). However, Acromyrmex laticeps and Acromyrmex niger ants did not differ in their susceptibility to infection when they carried their own *Pseudonocardia* or under *Pseudonocardia*-free conditions (Fig. 1C and D). In contrast, *A. echinatior* and Acromyrmex octospinosus ants reared under *Pseudonocardia*-free conditions (Fig. 1A and B) were 2.28 times more susceptible to infection than when they carried their own *Pseudonocardia*. When comparing a within-group of ants carrying their own *Pseudonocardia*, we found that all ants carrying their own *Pseudonocardia* differed in survival when challenged with Metarhizium (Wald = 13.79, df = 3, *P *= 0.003). Acromyrmex echinatior, *A. laticeps*, and A. niger were 1.8, 2.3, and 2.0 times more susceptible to infections by Metarhizium than *A. octospinosus* (Fig. 1).

### Metapleural glands and *Pseudonocardia* effects on ant defense.

**FIG 2:**
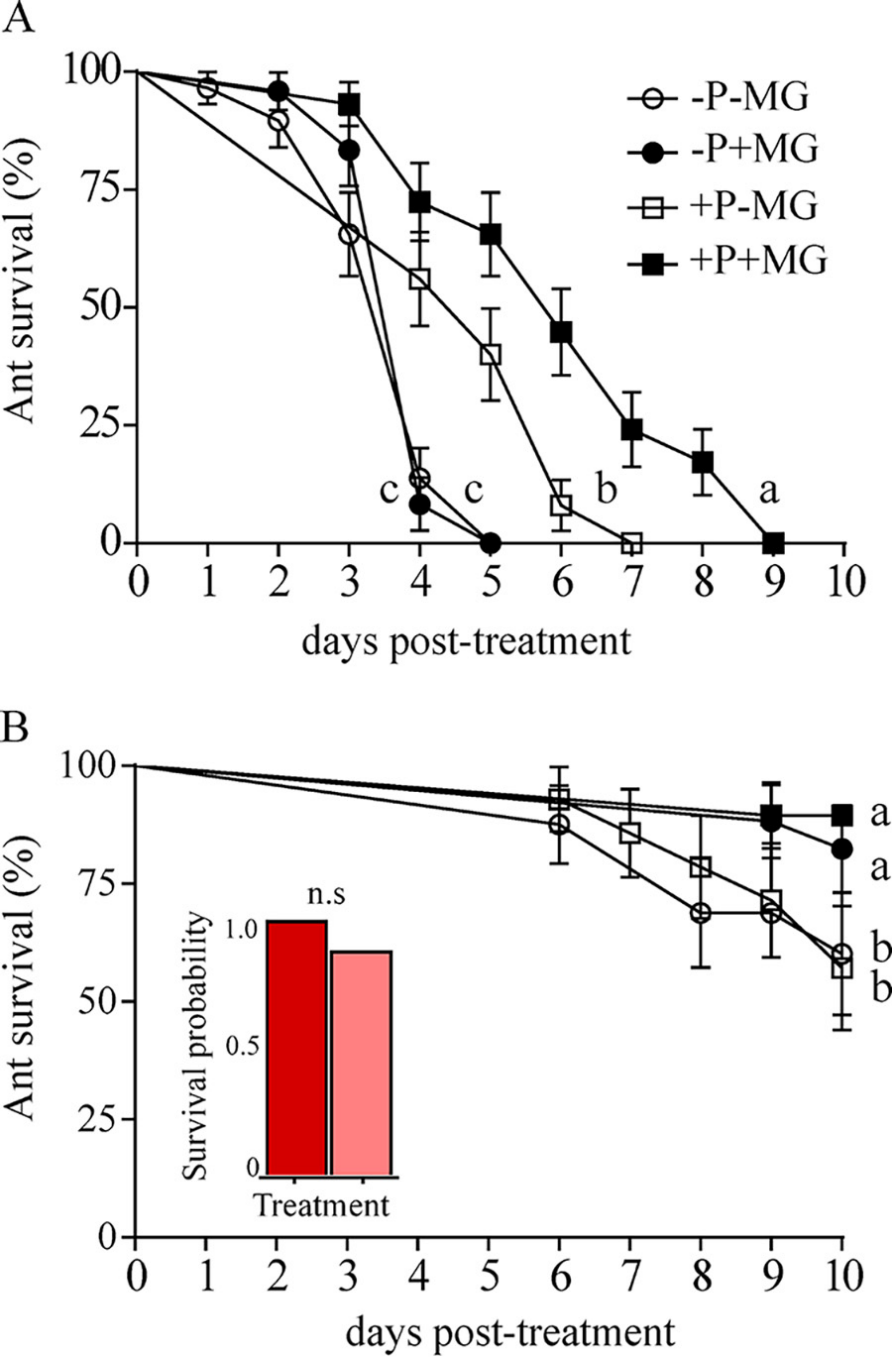
Metapleural glands and *Pseudonocardia* effects on ant defense. Metapleural glands and *Pseudonocardia* effects on *A. echinatior* workers infected with Metarhizium (A) and a control solution (B). Ants were reared under either *Pseudonocardia*-carrying conditions (+P) or *Pseudonocardia*-free conditions (−P) with either MGs sealed (−MG) or MGs open (+MG). A control solution was made of sterile deionized water + 0.01% Tween 20. Error bars represent standard error. Letters represent significant differences at a *P* value of <0.05 in pairwise comparisons using a Kaplan-Meier pairwise test. The inset graph shows the effects of the acrylic solution, which was used to block the MGs, on gaster-painted ants (red bar) and unpainted ants (pink bar).

Ant survival was significantly greater for control-treated ants than that for ants exposed to Metarhizium (Wald = 95.54.71, df = 1, *P < *0. 001). Interestingly, there were significant main effects within MG treatments (Wald = 9.27 df = 1, *P *= 0.002) and symbiotic condition (Wald = 7.37 df = 1, *P *= 0.007), but there were no significant differences for any interaction effect (*P > *0.05). *A. echinatior* ants with MGs open have a significantly lower risk (58.6%) of death than ants with MGs closed (Fig. 2A). In contrast, *A. echinatior* ants with a *Pseudonocardia*-free condition were 2.25 times more likely to die than those ants carrying *Pseudonocardia.* Metarhizium-treated ants had a 72.5% lower risk of death when associated with *Pseudonocardia* (Wald = 25.41, df = 1, *P < *0. 0001) and 35% lower risk when the MGs were open (Wald = 4.37, df = 1, *P *= 0.036) (Fig. 2A). When ants were exposed to the control solution, there were significant effects within MG treatments (Wald = 5.81, df = 1, *P *= 0.016), where ants with MGs open had a lower risk (72.9%) of death than those with MGs closed (Fig. 2B).

### Nonnative *Pseudonocardia* acquisition and bacterial coverage effects on ant individual susceptibility.

**FIG 3:**
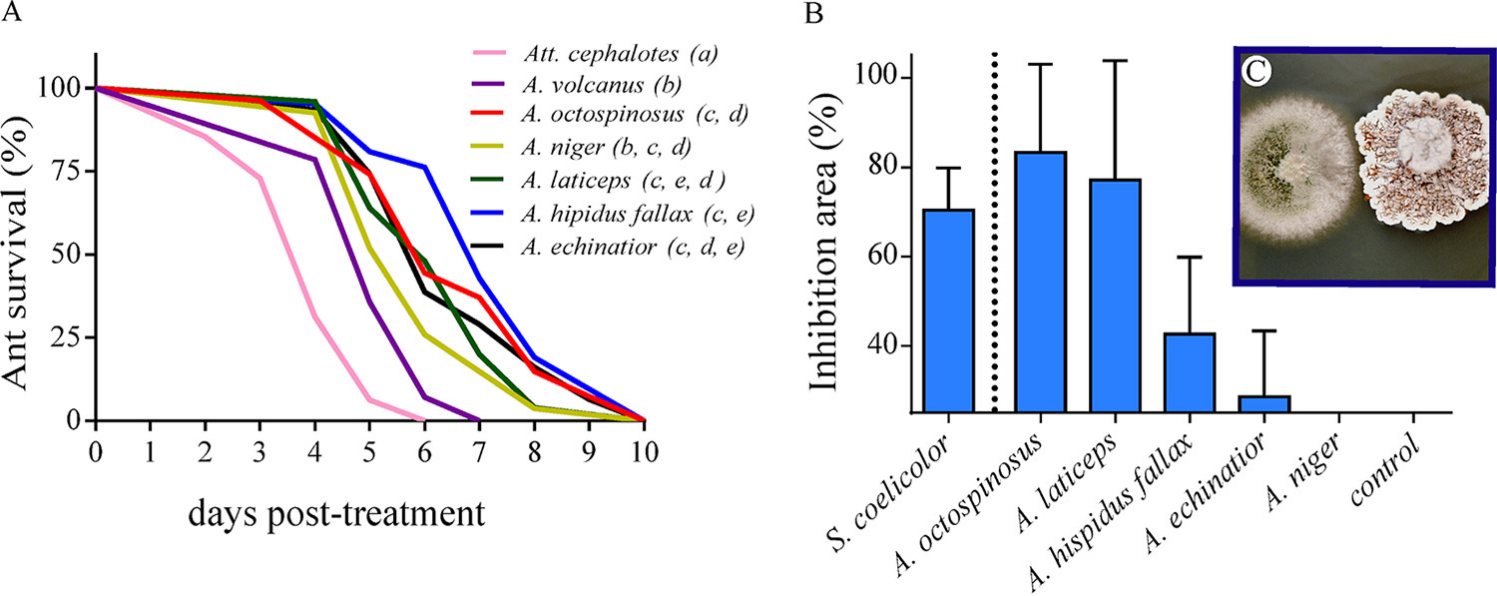
Nonnative *Pseudonocardia* acquisition and bacterial coverage effects on ant individual susceptibility. (A) Survivorship curves of *A. echinatior* workers carrying the *Pseudonocardia* ectosymbiont from different *Acromyrmex* species after being exposed to Metarhizium. Species names on the legend denote the ant host species from which each *Pseudonocardia* strain was derived. The *A. echinatior Pseudonocardia* is the native strain. *A. echinatior* ants raised by *A. cephalotes* ants are *Pseudonocardia* free. Letters represent significant differences from one treatment to another at a *P* value of <0.05 in pairwise comparisons using the Kaplan-Meier pairwise test. (B) Effects of different strains of *Pseudonocardia* isolated from different species of *Acromyrmex* ants on the conidial germination area of Metarhizium. Streptomyces coelicolor (a common soil-dwelling *Actinobacteria*) was used for comparative effects. (C) Micrograph illustrating the interactions between Metarhizium (left) and *Pseudonocardia* (right). Error bars represent standard error.

In the nonnative experiment, all ants exposed to the control solution survived, while worker ant mortality was significantly higher in the Metarhizium infection treatments, both for the control native pairing (Wald = 60.36, df = 1, *P < *0.0001) and for *A. echinatior* ants carrying *Pseudonocardia* from different ant species (conditions reared) (Wald = 62.9, df = 6, *P < *0.0001) (Fig. 3A). There was no correlation between the visible abundance of *Pseudonocardia* and ant survivorship (Wald = 0.02, df = 1, *P *= 0.885) nor between the phylogenetic clade of origin (see Fig. S1 in the supplemental material) for *Pseudonocardia* and ant survival (Wald = 1.77, df = 1, *P *= 0.83). Although pairwise comparisons of the survival distribution were variable within treatments (Fig. 3A), the hazard ratio for Metarhizium-treated ants was significantly higher for *A. echinatior* carrying either nonnative *Pseudonocardia* or carrying native (own colony) *Pseudonocardia* than that for *Pseudonocardia*-free *A. echinatior* ants (reference group) (see Table S1 in the supplemental material).

10.1128/mBio.01885-21.2 FIG S1 Maximum likelihood phylogenetic tree using elongation factor-Tu sequences of *Pseudonocardia* isolates. *Pseudonocardia* isolates from the experimental colonies are indicated by solid circles and other phylogenetically closely related attine-associated *Pseudonocardia*. The name of the *Pseudonocardia* isolates represents the attine species in which the bacteria was obtained, and GenBank accession number are shown in parenthesis. Clade (IV and VI) indicates the position of the *Pseudonocardia* isolates relative to previous studies (7, 8). The number before branch points supports bootstraps from 1,000 resampled datasets with values less than 50% not shown. Streptomyces griseus and Streptomyces sampsonii were used as outgroups. The scale bar indicates 0.02 change per nucleotide. Download FIG S1, DOCX file, 0.4 MB. Copyright © 2021 Bruner-Montero et al. 2021 Bruner-Montero et al. https://creativecommons.org/licenses/by/4.0/ This content is distributed under the terms of the Creative Commons Attribution 4.0 International license.

10.1128/mBio.01885-21.5 TABLE S1 Survivorship of Acromyrmex echinatior workers carrying different *Pseudonocardia* ectosymbionts after being treated with Metarhizium *anisopliae^a^* Table S1, DOCX file, 0.02 MB. Copyright © 2021 Bruner-Montero et al. 2021 Bruner-Montero et al. https://creativecommons.org/licenses/by/4.0/ This content is distributed under the terms of the Creative Commons Attribution 4.0 International license.

To compare the effects of acquiring nonnative *Pseudonocardia* on *A. echinatior*, we excluded *Pseudonocardia*-free ants from the Cox regression model and included only *A. echinatior* ants carrying their native *Pseudonocardia* (native) as a reference group. There were significant differences in ant survival within treatments (Wald = 21.32, df = 5, *P *= 0.001). The hazard ratio did not differ between *A. echinatior* ants carrying their own *Pseudonocardia* and *A. echinatior* ants carrying nonnative *Pseudonocardia* from the colonies of Acromyrmex hispidus fallax (*P *= 0.256), *A. laticeps* (*P *= 0.655), A. niger (*P *= 0.275), or *A. octospinosus* (*P *= 0.838). However, *A. echinatior* ants carrying *Pseudonocardia* from an Acromyrmex volcanus colony showed a significantly higher risk (2.4 times, *P *= 0.002) of death than *A. echinatior* ants carrying their own *Pseudonocardia*.

### Coverage of *Pseudonocardia* acquired from native and nonnative colonies in Acromyrmex echinatior.

Overall, there were significant effects of the *Pseudonocardia* coverage within ant species (*F*_5, 488_ = 161.93, *P < *0.0001), conditions reared (cross-fostered and native) (*F*_5, 488_ = 122.17, *P < *0.0001), and the interaction between condition reared and ant species (*F*_5, 448_ = 25.30, *P < *0.0001). A *post hoc* comparison showed that *Pseudonocardia* coverage was higher for *Acromyrmex* ants raised by their native colonies than that for *A. echinatior* ants raised by allospecific *Acromyrmex* ants (*P < *0.0001, for all). However, *Pseudonocardia* coverage between cross-fostered and native ants did not differ with *A. octospinosus* (*P *= 0.55) (see Fig. S2 in the supplemental material).

10.1128/mBio.01885-21.3 FIG S2 Graph comparing the difference in *Pseudonocardia* coverage (mean ± SE) on Acromyrmex echinatior workers carrying the *Pseudonocardia* ectosymbiont from different leaf-cutter ant species (cross-fostered) and when these *Acromyrmex* species are reared by their conspecific nestmates (native colony). The bar within the dashed lines represents *A. echinatior* ants raised only by their own colony, and *A. cephalotes*, which naturally does not carry *Pseudonocardia*, was used as negative control of *Pseudonocardia* acquisition. Download FIG S2, DOCX file, 0.07 MB. Copyright © 2021 Bruner-Montero et al. 2021 Bruner-Montero et al. https://creativecommons.org/licenses/by/4.0/ This content is distributed under the terms of the Creative Commons Attribution 4.0 International license.

### *Pseudonocardia-*Metarhizium *in vitro* challenges.

There was a significant effect of *Actinobacteria* strain on the reduction of *Metarhizium* mycelial growth area (*F*_5, 29_ = 4.31, *P *= 0.0046) and on the reduction of the conidium germination area (*F*_5, 29_ = 19.21, *P < *0.0001). *Post hoc* comparisons revealed that Streptomyces coelicolor significantly reduced mycelial growth (*P < *0.05) compared with the rest of the *Actinobacteria* treatments, except for the strain of *Pseudonocardia* isolated from A. niger (*P *= 0.107). *Pseudonocardia* from *A. laticeps*, *A. octospinosus*, and S. coelicolor showed a greater reduction of conidium germination area than the rest of the *Actinobacteria* (*Pseudonocardia* strains from *A. echinatior*, A. niger, and *A*. *hispidus fallax*; *P < *0.001, for all cases) (Fig. 3B). However, S. coelicolor and the *Pseudonocardia* strains isolated from *A. laticeps* did not differ significantly (*P *= 0.25), nor did *Pseudonocardia* strains from A. niger and *A. echinatior* (*P *= 0.08). The conidium germination coverage did not show significant differences between treatments with *Pseudonocardia* from *A. hispidus fallax* and treatments with strains from both *A. laticeps* (*P *= 0.07) and *A. echinatior* (*P *= 0.82) (Fig. 3B).

### MALDI imaging.

**FIG 4:**
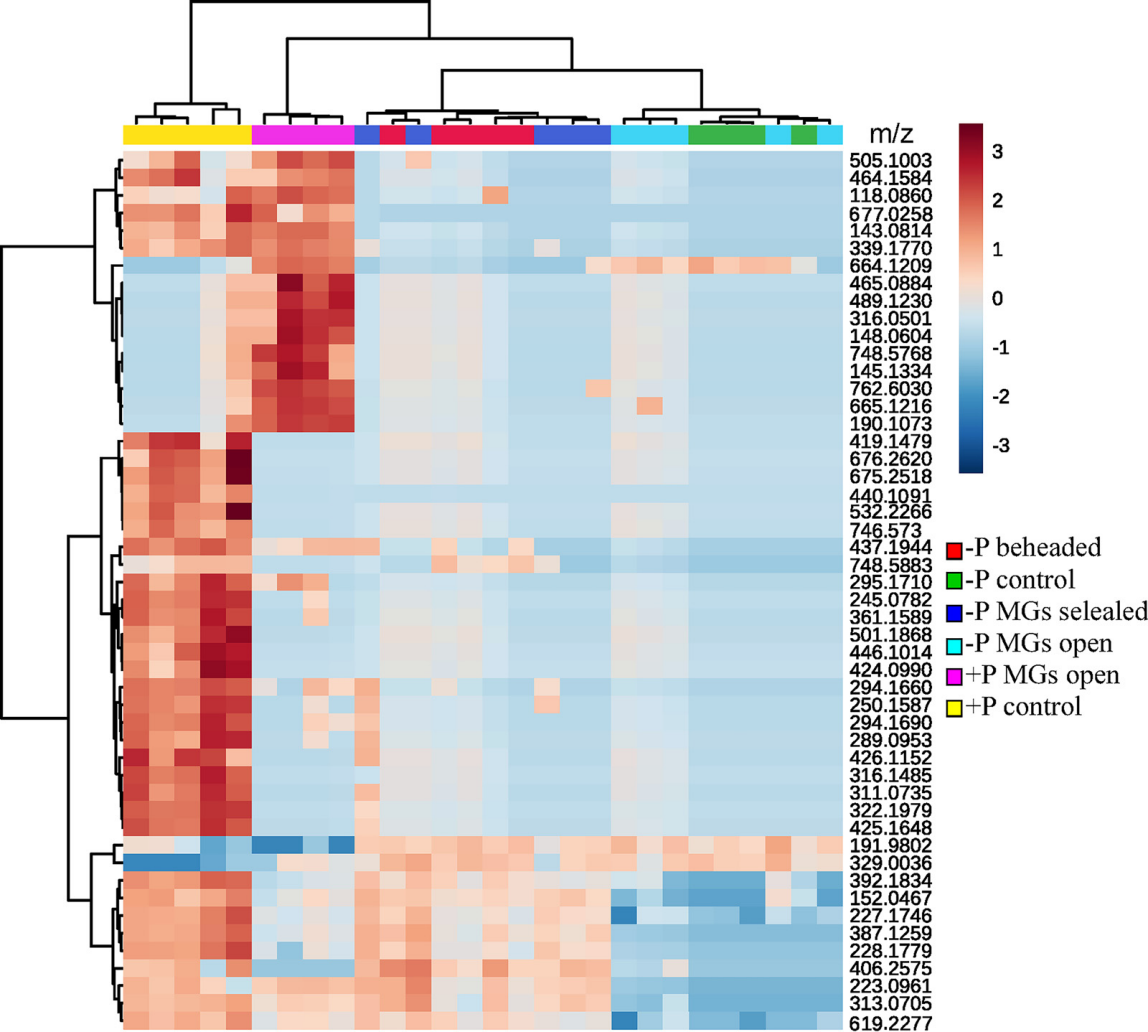
*In situ* imaging mass spectrometry of *Acromyrmex* ants. Heat map shows the 50 top putative metabolites (row) of importance. Metabolites were selected based on an ANOVA test between treatments and clustered by similarities. Ants were reared under either *Pseudonocardia*-carrying conditions (+P) with MG opened (pink) and MG opened control (yellow) or *Pseudonocardia*-free conditions (−P) with MGs opened (light blue), MGs sealed (blue), beheaded (red), and MG opened control (green). Metarhizium-treated ants are denoted by the inset green square. Control denotes uninfected treatments. The color key indicates metabolite relative intensity (blue, lowest; red, highest).

MALDI imaging detected 41,724 peaks with an average of 1,490.1 peaks per sample. A heat map revealed that the distribution of the putative metabolites associated with ants carrying *Pseudonocardia* with MGs open and infected with Metarhizium (Fig. 4). Partial least-squares discriminant analysis (PLS-DA) data clearly showed discrimination between groups, particularly between *Pseudonocardia*-carrying treatments and *Pseudonocardia*-free treatments (see Fig. S3 in the supplemental material).

10.1128/mBio.01885-21.4 FIG S3 PLS-DA of the profile of metabolites associated with the ants exoskeleton treated with Metarhizium (inset green square) and control treatments. Dots on the graph represent a single ant per treatment clustered by a 95% confidence interval. Ants were reared under either *Pseudonocardia*-carrying conditions (+P) with MG opened (pink) and MG opened control (yellow) or *Pseudonocardia*-free conditions (−P) with MGs opened (light blue), MGs sealed (blue), beheaded (red), and MG opened control (green). Control denotes uninfected treatments. Download FIG S3, DOCX file, 0.08 MB. Copyright © 2021 Bruner-Montero et al. 2021 Bruner-Montero et al. https://creativecommons.org/licenses/by/4.0/ This content is distributed under the terms of the Creative Commons Attribution 4.0 International license.

## DISCUSSION

Our study explores the epidemiological and chemical dynamics involved in *Pseudonocardia*-derived protection against a generalist entomopathogen (Metarhizium *anisopliae*) in *Acromyrmex* leaf-cutter ants. Overall, our results indicated that the ecosymbiont *Pseudonocardia* helps confer increased protection to ant workers from entomopathogenic diseases. Specifically, we experimentally show a substantial reduction in Metarhizium-induced mortality in *Acromyrmex* workers with *Pseudonocardia.* Furthermore, our findings that (i) *Pseudonocardia-*carrying ants produced more compounds in response to Metarhizium infection than *Pseudonocardia*-free ants, (ii) *in vitro Pseudonocardia* reduced Metarhizium conidium germination area, and (iii) nonnative *Pseudonocardia* still conferred protection to *A. echinatior* ants against Metarhizium, despite reduced physical coverage of workers in some switches, support the hypothesis that *Pseudonocardia-*derived antifungal compounds are involved in helping protect ants from an entomopathogenic fungus. Our results reflect an apparent complex evolutionary dynamic between the specialized chronic parasite *Escovopsis* and a cosmopolitan insect-pathogenic fungus and a selective pressure for acquiring *Pseudonocardia* partners to protect their hosts from pathogen outbreaks.

It is notable that when *A. echinatior* and *A. octospinosus* ants were reared under *Pseudonocardia*-free conditions, they were more susceptible to Metarhizium, while *A. laticeps* and A. niger ants had a similar susceptibility to Metarhizium under either condition (i.e., with or without *Pseudonocardia*). This result indicates that an ant’s individual susceptibility to Metarhizium and the level of *Pseudonocardia*-derived protection can differ between *Acromyrmex* species. However, interpreting this variation requires an understanding of the interaction between host and symbiont genotypes and natural variation in resistance against Metarhizium (48–50). Indeed, natural variation in resistance to Metarhizium has been observed among colonies of *A. echinatior* (51) and other social insects, such as termites (52). Another source of natural variation against pathogens can be derived from different symbiont genotypes. For example, distinct strains of the endosymbiotic bacterium *Wolbachia* can provide different levels of protection against several pathogenic RNA viruses in *Drosophila* flies (53). Variable protection among distinct symbiont genotypes has also been reported in pea aphids by the defensive symbiont Hamiltonella defensa against parasitoid wasps.

Experimental manipulation of the presence/absence of MGs and *Pseudonocardia* symbiont on ants show that Metarhizium-treated *A. echinatior* ants had a larger reduction in pathogen-induced mortality (72.5%) when associated with *Pseudonocardia* than when the MGs were open (35%). Furthermore, *A. echinatior* ants that did not carry *Pseudonocardia* were at an increased risk of death regardless of whether their MGs were open or sealed. These results provide further support that *Pseudonocardia* helps protect workers from entomopathogens, such as Metarhizium, and that the antibiotics secreted by *Pseudonocardia* appear to play a larger role in attenuating diseases than MG-secreted antibiotics in some *Acromyrmex* ants. Ants can selectively apply MG secretions to themselves (self-grooming) and other nestmates (allogrooming) as a social prophylaxis strategy to reduce the transmission of diseases within the colony (36, 54–58). Several studies have demonstrated that MG secretions display antimicrobial activity in attine ants (55, 59–61) and other ant species (62, 63). However, it has been shown in leaf-cutter ants (i.e., *Atta* and *Acromyrmex*) that the role of MG use differs quantitatively and qualitatively in the spectrum of compounds secreted between species, colonies, and worker caste (39, 63, 64). It was argued recently that these differences may be a consequence of the trade-off between MG secretions and *Pseudonocardia* in the production of antimicrobial metabolites (39, 57, 60, 61). While *Acromyrmex* ants depend on producing specialized *Pseudonocardia-*derived antibiotics for suppressing the fungal garden pathogen *Escovopsis*, it seems that *Atta* ants compensate for the lack of *Pseudonocardia* by producing broad-spectrum antimicrobials in their MGs. According to this compensatory hypothesis and our data, it is possible that there is a high selection pressure in *Acromyrmex* ants to select *Pseudonocardia* strains capable of producing complementary compounds, in addition to those that inhibit *Escovopsis*, that suppress generalized pathogens in compensation for reduced protective effects of MG secretions. Further research should be undertaken to investigate the chemical and physiological relationship between the bioactive compounds derived from MG secretions and *Pseudonocardia* across attine ants, particularly in those that lack the association with *Pseudonocardia*, such as *Sericomyrmex*.

One interesting finding is that the antifungal protective effect of *Pseudonocardia* was extended to *A. echinatior* ants even when they acquired nonnative *Pseudonocardia* from geographically distant species. This finding suggests that the acquisition of nonnative *Pseudonocardia* may not affect individual performance (i.e., survival) of the ants and further shows evidence that horizontal transmission may be plausible as previous phylogenetic studies argue (22, 47). These findings are consistent with Armitage et al. (83) and Andersen et al. (65) who reported that sympatric Panamanian *Acromyrmex* ants (*A. echinatior*, *A. octospinosus*, and *A. volcanus*) can exchange their *Pseudonocardia* strains in the laboratory.

It has been reported that colonies of sympatric Panamanian *Acromyrmex* ants are colonized predominantly by only one of two possible *Pseudonocardia* lineages (clade IV and VI), indicating prevalence and stability within ant-*Pseudonocardia* populations (22, 45, 46, 65). However, these previous studies were focused on a small area around the Panama Canal Zone, Gamboa, Panama. In contrast, one interesting finding in our study is that *A. echinatior* ants, which carry *Pseudonocardia* strain VI, were similarly resistant to Metarhizium when they acquired nonnative *Pseudonocardia* strains IV and VI from both South American and Central American *Acromyrmex* species. It is possible that both clades are equally protective against pathogens. Consequently, a possible explanation for this persistence might be that these strains (clade IV and VI) are coadapted to *Acromyrmex* ants, in accordance with the data of Cafaro et al. (2011) (22), and are under high selection pressure to combat pathogens (24, 45, 46, 66). A future approach for understanding the evolutionary dynamic between ant-associated *Pseudonocardia* and ant-infecting pathogen diversity is the incorporation of whole-genome analysis across regional and local levels.

When *A. echinatior* pupae were cross-fostered with allospecific caregiver workers, the ants acquired a lower abundance of *Pseudonocardia* in contrast to *A. echinatior* ants reared by their own colony. These findings indicate a certain degree of ant-*Pseudonocardia* affinity, which was also found by Armitage et al. (2011) and Andersen et al. (2015). We found no correlation between the relative visible coverage of *Pseudonocardia* and ant mortality. These results suggest that the bacterial coverage does not explain the susceptibility to Metarhizium. However, it has been demonstrated that the presence of *Escovopsis* within fungus gardens can result in corresponding increases in *Pseudonocardia* coverage on workers (41, 61). It is difficult to interpret whether the filaments of the bacterium are physically protecting the ant body because we observed similar beneficial effects in all nonnative *Pseudonocardia* transmitted to *A. echinatior* ants regardless of the bacterial coverage. However, our *in vitro* and *in situ* results suggest that chemical protection derived from *Pseudonocardia* plays a significant role in protection.

The chemical protection of *Pseudonocardia* can differ when the bacterium is associated with the ant cuticle or when it is grown under *in vitro* conditions. For example, we noticed that nonnative *Pseudonocardia* strains from *A. hispidus fallax* and A. niger conferred protection to *A. echinatior* ants. However, these *Pseudonocardia* strains differed in their suppressing effect on the spore germination of Metarhizium under *in vitro* conditions. These differences need to be treated with caution because the cuticle of the ant represents a limited nutritional environment where cuticular crypts supply nutrients to *Pseudonocardia* (67–69). In contrast, a synthetic medium provides a nutritionally rich environment. How these different conditions affect the expression of antimicrobial compounds against Metarhizium is unknown, but it is more likely that the production of antibiotics occurs under stress conditions, such as nutrient depletion (70–72). Furthermore, it is likely that *Pseudonocardia* responds to the antimicrobial demands of its ant host (73).

Within *Actinobacteria*, the family *Pseudonocardiaceae* is recognized for producing bioactive antimicrobial compounds. Consequently, it might be expected that *Pseudonocardia* produces bioactive secondary metabolites to protect the ants against other pathogens besides *Escovopsis*. There have indeed been many studies showing that the metabolic potential of *Pseudonocardia* ranges from a broad spectrum to a narrow spectrum of antimicrobial activity. For example, Barke et al. (2010) isolated a nystatin-like polyene from a colony of *A. octospinosus* with weak activity against Candida albicans and *Escovopsis* (74). In contrast, Meirelles et al. (2014) showed that several *Pseudonocardia* strains isolated from *Trachymyrmex* ants may have a generalized inhibitory activity against *Escovopsis* (38). Other previous studies have documented similar activities (41, 42, 75–77). Taken together, these studies suggest that there is high variability in antimicrobial activity by *Pseudonocardia* (42).

We found a high number of putative metabolites that might have a defensive role against the insect pathogen Metarhizium. However, characterizing the identity of these metabolite signals, expressed across different treatments, is difficult given the apparent complexity in the chemical response. Nevertheless, the results of the *Pseudonocardia*-Metarhizium challenges and MALDI-MSI support the conclusion that the protective benefits we observed are likely conferred by chemical protection. Additionally, the PLS-DA and heat map reveal that ants associated with *Pseudonocardia* secrete a distinct and more complex profile of compounds than ants lacking *Pseudonocardia* and not challenged with Metarhizium. This result indicates that the production of different compounds is down- and upregulated during Metarhizium infections. Because Metarhizium conidial adhesion to the insect cuticle is necessary for infection (78), it is possible that the high diversity of biomolecules is acting synergistically and contingently to reduce conidial germination rate. Insect-associated microbial symbionts depend on secondary metabolite products to mediate the host-symbiont association and inhibit both host-targeting pathogens and potential symbiont competitors. Therefore, it is not surprising that *Pseudonocardia* responded chemically to Metarhizium. In general, it seems that attine ants prevent and ameliorate disease using a complex arsenal of bioactive compounds whose functions need to be further studied.

This research extends our knowledge of *Pseudonocardia*-mediated protection in *Acromyrmex* ants. The results of this research support the idea that *Pseudonocardia* promotes ant resistance to pathogens by inducing an antimicrobial coating effect on the ant exoskeleton. It would be interesting to assess the collective effect (social immunity) of *Pseudonocardia* on the colony level. Mature *Acromyrmex* ant colonies maintain thousands of workers that harbor mutualistic *Pseudonocardia*; therefore, further experimental investigations are needed to estimate the amount of secondary metabolites secreted in the colony. As an analogy to the health care industry in which antimicrobial agents are applied to the surface of a material to reduce the growths of microorganisms, we suggest that *Pseudonocardia* acts as an antimicrobial surface agent promoting colony health by providing biotherapeutic medication on the individual and colony level.

## MATERIALS AND METHODS

### Study species.

Seven species of leaf-cutter ants were used in our experiments. Four species that coexist sympatrically in Central America were used, as follows: Acromyrmex echinatior (AL050505-11; Panama), *Acromyrmex octospinosus* (ST04116-01; Panama), *Acromyrmex volcanus* (CR2014; Costa Rica), and Atta cephalotes (AL050513-22; Costa Rica). The following three South American species were used: *Acromyrmex laticeps* (UGM030330-05; Argentina), *Acromyrmex hispidus fallax* (UGM030327-02; Argentina), and *Acromyrmex niger* (CC030327-02; Argentina). The *Acromyrmex* species used in this study carry abundant visible *Pseudonocardia* on their exoskeleton. Colonies were kept in large plastic containers in the Microbial Science Building at the University of Wisconsin-Madison.

### Cross-fostering technique.

We used a cross-fostering technique to manipulate *Pseudonocardia* acquisition in which medium-sized pupae from a leaf-cutter ant colony were removed and raised by caregiver ants from either conspecific (own colony) or allospecific (*Atta* or *Acromyrmex*) workers. Using this approach, we generated aposymbiotic (i.e., *Pseudonocardia* free) workers and switched ant-*Pseudonocardia* combinations (i.e., switch *Pseudonocardia* symbionts between ant hosts). Members of the genus *Atta* do not have *Pseudonocardia* on their exoskeleton, and so *Atta* caregiver workers do not transmit the ectosymbiont (43). These *Pseudonocardia*-free ants were used as controls (see below).

### Individual-level susceptibility across *Acromyrmex*.

To evaluate whether *Pseudonocardia* helps protect different species of *Acromyrmex* ants from infection by Metarhizium, we examined the susceptibility of four *Acromyrmex* species under *Pseudonocardia*-free conditions. We generated *Pseudonocardia-*free ants by rearing pupae of *Acromyrmex* ants with workers of *A. cephalotes*. For the experiment, we set up two subcolonies (*n* = 8) per species consisting of a weigh boat placed in a petri plate with a ring of moist cotton to maintain the humidity, 3 g of *A. cephalotes* fungal garden, and 50 focal medium-size pupae of either *A. echinatior*, A. niger, *A. laticeps*, or *A. octospinosus*. One subcolony of each species was placed with either 70 workers (15 major, 25 medium, and 30 minima workers) of its own species (own colony) or *A. cephalotes.* We monitored each subcolony every 3 days to remove and replace dead caregiver workers and fungus garden rejected by the ants; rejected pupae were not replaced. Unless otherwise specified, we used the fungus garden from *A. cephalotes* in all our experiments to control for any nutritional differences among gardens that may have affected the ant performance (79). After 15 to 18 days posteclosion, all ants were each placed into an individual Petri plate with a ring of moist cotton. Then, the propleural plate of each ant was inoculated with Metarhizium. *M. anisopliae* was applied using 1 μl of ca. 1.00 × 10^7^ conidia ml^−1^ suspension + 0.01% Tween 20 by using a micropipette. These treatment setups were repeated and inoculated with a control solution of sterile, deionized water + 0.01% Tween 20. The survival of the ants was monitored every 24 h posttreatment for 10 days.

*M. anisopliae* is a broad-spectrum insect pathogen, known to infect many insect species (80). In our research, we were interested in the generalized capacity of Metarhizium to infect many different arthropods. The *M. anisopliae* strain used in our experiments was isolated from dead bee workers of Apis mellifera and showed the ability to infect ants. For the experiments, conidia were taken from recently sporulating cultures on potato dextrose agar and suspended in a solution of sterile deionized water containing 0.01% Tween 20. The conidial concentration was quantified using a hemocytometer and diluted to a concentration of ca. 1.00 × 10^7^ conidia ml^−1^.

### Metapleural gland and *Pseudonocardia* effects on ant defense.

To test the effects of the compounds produced by the MGs and *Pseudonocardia* on ant susceptibility to Metarhizium, we reared *A. echinatior* ants with or without *Pseudonocardia* and MGs open or MGs sealed. To produce *Pseudonocardia*-free ants, we cross-fostered *A. echinatior* pupae with *A. cephalotes* workers as described above. We set up two subcolonies as described above containing 5 g of fungal garden from *A. cephalotes*, 60 focal pupae from *A. echinatior*, and 70 workers of either *A. echinatior* or *At. cephalotes.* All ants were monitored, and worker and fungus garden were replaced as mentioned above. After 15 to 18 days posteclosion, we blocked the MG of the ants reared under both the *Pseudonocardia*-free condition (*n* = 30) and *Pseudonocardia*-carrying condition (*n* = 30) by applying a harmless acrylic solution with a paintbrush (Fig. 2, inset graph). All ants (*Pseudonocardia*-free ants with MG open and sealed and *Pseudonocardia*-carrying ants with MG open and sealed) were each placed into an individual Petri plate with a ring of moist cotton. Ants were then treated with Metarhizium or control solution and monitored as described above.

### Nonnative *Pseudonocardia* acquisition and coverage effects on ant susceptibility.

To examine whether the acquisition of nonnative *Pseudonocardia* affects the individual susceptibility of the ants to Metarhizium, we manipulated *Pseudonocardia* acquisition by rearing pupae from a colony of *A. echinatior* with ants from seven leaf-cutter ant species (*A. echinatior*, *A. octospinosus*, *A. hispidus fallax*, A. niger, *A. laticeps*, *A. volcanus*, and *A. cephalotes*) that significantly differ in the visible abundance of *Pseudonocardia* on their exoskeleton, ranging from not visible to highly abundant (22, 44, 57).

For this experiment, we set up one subcolony (*n* = 7) per species as described above containing 6 g of fungal garden; 50 focal medium-size pupae from *A. echinatior*; and 70 workers of either *A. echinatior* (own colony), *A. octospinosus*, *A. hispidus fallax*, A. niger, *A. laticeps*, *A. volcanus*, or *A. cephalotes.* All ants were monitored, placed in individual subcolonies, and treated with Metarhizium or the control solution as mentioned previously. Before infecting the ants, we scored *Pseudonocardia* coverage via stereomicroscope using the scale designed by Poulsen et al. (44), which scores from 0 (no visible *Pseudonocardia*) to 12 (ant cuticle is totally covered with *Pseudonocardia*).

### *Pseudonocardia-*Metarhizium *in vitro* challenges.

To experimentally examine the antifungal properties of *Pseudonocardia* against Metarhizium, we performed pairwise bioassays in which pure cultures of *Pseudonocardia* were challenged with Metarhizium isolates. For each microbial bioassay, we inoculated *Pseudonocardia* in the center of a Petri plate (100 mm by 15 mm) containing yeast malt extract agar (YMEA) and allowed it to grow for 10 weeks. The Metarhizium isolate was then inoculated 1 cm from the *Pseudonocardia* area using a suspension solution of 1 μl of ca. 1.5 × 10^6^ conidia ml^−1^. At 8 days postfungal inoculation, each Petri plate was photographed and the mycelial growth area and conidia germination coverage relative to the mycelial growth area were measured using the software ImageJ (http://rsbweb.nih.gov/ij/). We tested the inhibitory proprieties of *Pseudonocardia* using pure isolates from the experimental colonies *A. echinatior*, *A. octospinosus*, *A. hispidus fallax*, A. niger, and *A. laticeps*. We also measured the inhibitory properties of Streptomyces coelicolor, a common soil-dwelling *Actinobacteria* (*n* = 5 to 8 per pairing). As a positive control, we challenged Metarhizium against antifungal disks soaked in nystatin with a concentration of 10,000 units ml^−1^. *Pseudonocardia* was isolated from worker cuticles using a method from Cafaro and Currie (2005) (47). Despite extensive efforts, we were unable to isolate *Pseudonocardia* from the colony of *A. volcanus* using this method.

### MALDI-Orbitrap imaging.

We then assessed whether *Pseudonocardia* produces *in vivo* compounds that inhibit Metarhizium. To do so, 28 medium-size pupae were collected randomly from the top of an *A. echinatior* fungal garden. The ants were split into two groups, as follows: *A. echinatior* ants (*n* = 19) reared under a *Pseudonocardia*-free condition and *A. echinatior* ants (*n* = 9) reared with their own *Pseudonocardia* using the cross-fostering approach in the experiment above. Fifteen days posteclosion, each ant was placed in a single Petri plate with a small piece moist cotton. To reduce other sources of secondary metabolites, we tested *Pseudonocardia*-free ants under the following treatments: removal of the ant’s head (*n* = 5), sealing of the MGs (*n* = 5), and keeping MGs open (*n* = 5). The ant’s propleural plate was then inoculated with Metarhizium using the following treatments: *Pseudonocardia*-free ants (beheaded, MG sealed, and MG open) and *Pseudonocardia*-carrying ants with MG open (*n* = 5). Two groups, namely, *Pseudonocardia*-free ants with MG open (*n* = 4) and *Pseudonocardia*-carrying ants with MG open (*n* = 4), were inoculated with sterile-deionized water as a control. The other ants were inoculated with a 1 μl of ca. 1.00 × 10^7^ conidia ml^−1^ suspension of *M. anisopliae + *0.01% Tween 20. Twenty-four hours postinfection, ants were collected and stored at −20°C. All ants were transported to the School of Pharmacy at the University of Wisconsin-Madison for mass spectrometry imaging (MSI) analysis (84).

### DNA extraction, sequencing, and phylogenetic analysis.

DNA from was extracted, and a partial length sequence of the nuclear elongation factor gene (EF-Tu) was amplified using primers 52F and 920R (46). All sequences were aligned and a maximum likelihood-based phylogeny was generated. Twenty-six sequences in the GenBank database from previous studies were added to the analysis for comparison (see electronic supplemental material).

### Statistical analysis.

We analyzed ant survivorship using a Cox regression model. The Cox regression model produces a survival function (i.e., hazard) that predicts the probability of death associated with a variable(s) at a specific time. The hazard ratio was estimated for the experiments using the categorical variables as follows: fungal treatment (Metarhizium versus control), symbiotic condition (*Pseudonocardia*-carrying ants and *Pseudonocardia*-free ants), condition reared (*Pseudonocardia-*donor ant species, namely, *A. echinatior*, *A. octospinosus*, *A. volcanus*, *A. laticeps*, *A. hispidus fallax*, A. niger, and *A. cephalotes*), *Pseudonocardia* clade (clade V versus clade IV), and visible abundance (i.e., coverage) of *Pseudonocardia* was used as a covariable. The relative risk reduction parameter was estimated to quantify the relative decrease in the risk of death (see Table S1). A full model was performed to estimate the main effects and their interaction effects. Separately, Metarhizium-treated ants were analyzed to estimate and compare the effects of resistance across the treatments. To compare within treatments, we used the Kaplan-Meier analysis. A two-way analysis of variance (ANOVA) was used to analyze the abundance of *Pseudonocardia* between ant species and condition reared (cross-fostered and native) and the interaction effect. For *Pseudonocardia*-Metarhizium challenges *in vitro*, a one-way ANOVA was used to analyze mycelium growth area and conidium germination area. A *post hoc* comparison between treatments was made using the Tukey’s honestly significant difference (HSD) test. All statistical analyses were performed in SPSS v. 22.

Mass spectrum results of the samples were statistically analyzed using MetaboAnalyst 3.0 (http://www.metaboanalyst.ca) (81). The low-intensity peaks (noise) were replaced by half of the minimum positive values of the data set, and the standard deviation option was applied to filter data as recommended by Hackstadt and Hess (85). The data were normalized using a reference control sample (control, uninfected *Pseudonocardia*-carrying ants) and transformed using a generalized logarithm transformation. The relative change of the masses between treatments was applied with the auto-scaling option. A heat map and principal-component analysis (PCA) were constructed to explore the dimension of the data set, followed by a partial least-squares discriminant analysis (PLS-DA) to reduce dimensionality.

### Data availability.

Raw sequencing data have been deposited in the GenBank database (accession numbers OL630658 to OL630663). The data sets generated during the current study are available from the corresponding authors on request.

10.1128/mBio.01885-21.1 TEXT S1 Contains expanded materials and methods of procedures, including DNA extraction, phylogenetic analysis methods, and MALDI-Orbitrap imaging. In addition, it contains expanded references for methods. Download Text S1, DOC file, 0.05 MB. Copyright © 2021 Bruner-Montero et al. 2021 Bruner-Montero et al. https://creativecommons.org/licenses/by/4.0/ This content is distributed under the terms of the Creative Commons Attribution 4.0 International license.
