# Supplementary material for: Symbiont-Mediated Protection of Acromyrmex Leaf-Cutter Ants from the Entomopathogenic Fungus Metarhizium anisopliae
Source: mBio. 2021 Dec 21;12(6):e01885-21. doi: 10.1128/mBio.01885-21 (PMC8689564; doi:10.1128/mBio.01885-21)
Supplement: FIG S1 [file mbio.01885-21-sf001.docx]

*
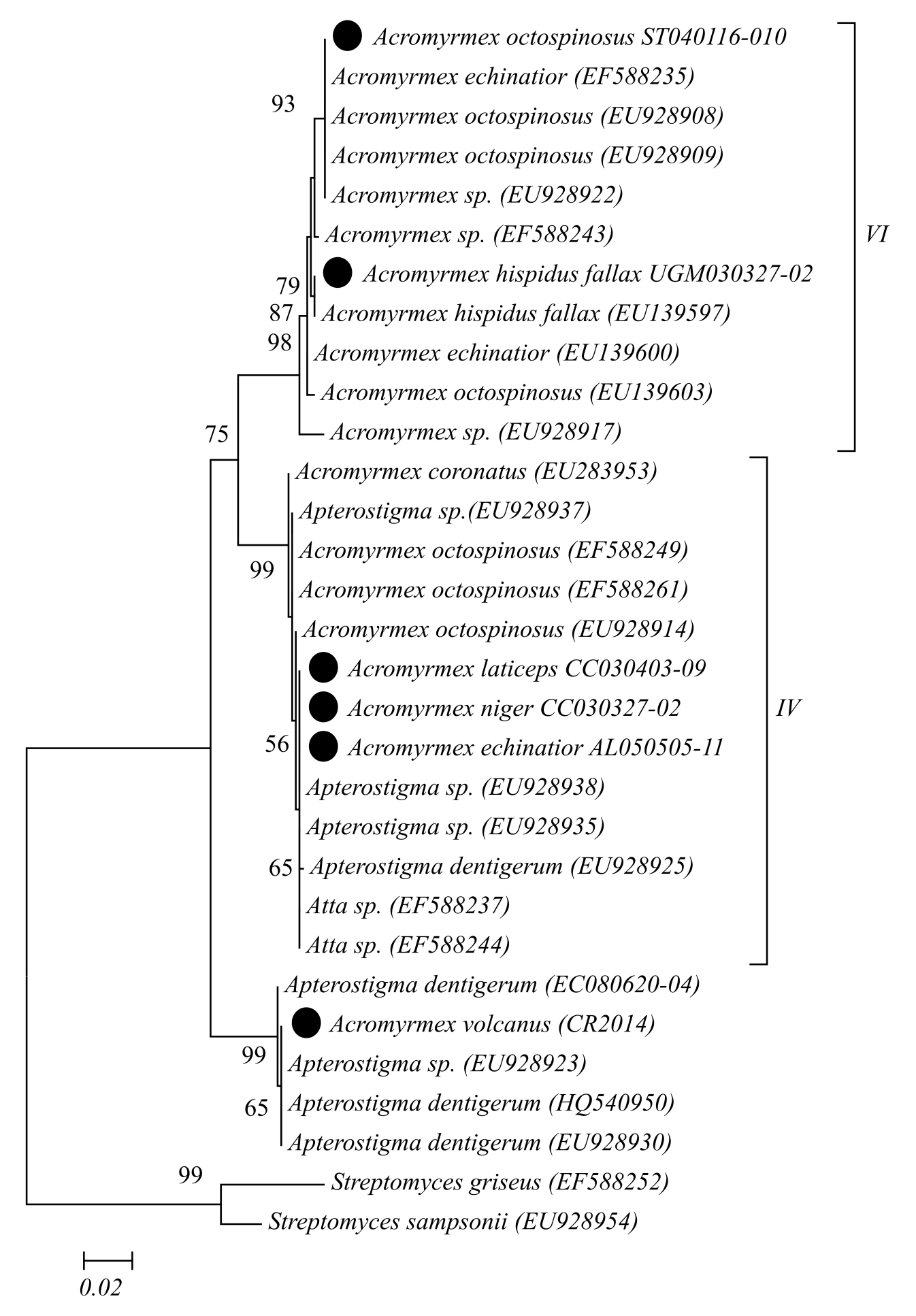
*

Figure S1. Maximum likelihood phylogenetic tree using elongation factor-Tu sequences of *Pseudonocardia* isolates. *Pseudonocardia* isolates from the experimental colonies are indicated by solid circles and other phylogenetically closely related attine-associated *Pseudonocardia*. Name of the *Pseudonocardia* isolates represents the attine species in which the bacteria was obtained and GenBank accession number are shown in parenthesis. Clade (IV and VI) indicate the position of the *Pseudonocardia* isolates relative to previous studies [[7](#_ENREF_7), [8](#_ENREF_8)]. The number before branch points support bootstrap from 1000 resampled datasets with values less than 50% not shown. *Streptomyces griseus* and *S. sampsonii* were used as outgroup. The scale bar indicates 0.02 change per nucleotide.
