# Supplementary material for: Symbiont-Mediated Protection of Acromyrmex Leaf-Cutter Ants from the Entomopathogenic Fungus Metarhizium anisopliae
Source: mBio. 2021 Dec 21;12(6):e01885-21. doi: 10.1128/mBio.01885-21 (PMC8689564; doi:10.1128/mBio.01885-21)
Supplement: TABLE S1 [file mbio.01885-21-st001.docx]

**Table S1.** Survivorship of *Acromyrmex echinatio*r workers carrying different *Pseudonocardia* ectosymbiont after being treated with *Metarhizium anisopliae^a^*

| Condition^b^ | N^c^ | *Pseudonocardia* cover^d^ | Wald^e^ | df^f^ | *P*^g^ | Exp(B)^h^ | RRR^i^ | 95.0% CI  for Exp.(B)^j^ | | |
| --- | --- | --- | --- | --- | --- | --- | --- | --- | --- | --- |
|  |  |  |  |  |  |  |  | Lower | Upper | |
| Treatment |  |  | 65.34 | 6 | ** |  |  |  | |  |
| *A. hispidus fallax* | 21 | 2.83 | 51.79 | 1 | ** | 0.108 | 89.2 | 0.05 | | 0.19 |
| *A. octospinosus* | 54 | 10.26 | 26.31 | 1 | ** | 0.158 | 84.6 | 0.07 | | 0.31 |
| *A. echinatior* | 31 | 8.06 | 27.20 | 1 | ** | 0.161 | 83.9 | 0.08 | | 0.32 |
| *A. laticeps* | 25 | 6.12 | 29.87 | 1 | ** | 0.182 | 81.8 | 0.09 | | 0.33 |
| *A. niger* | 27 | 2.00 | 32.33 | 1 | ** | 0.227 | 77.3 | 0.13 | | 0.37 |
| *A. volcanus* | 28 | 5.43 | 11.56 | 1 | * | 0.387 | 61.3 | 0.22 | | 0.66 |

1. Results representing the Cox Regression analysis of worker survival data; *Pseudonocardia*-free workers (n= 48) were used as reference group.
2. Denote the caregiver species in which *A. echinatior* pupae acquired the *Pseudonocardia.*
3. Number of ants treated.
4. Average of the abundance of visible *Pseudonocardia.*
5. Wald statistic for the treatments.
6. Degree of freedom for the Wald statistic.
7. Significance level for the Wald statistic; ** and * represent significant differences at *p* < 0.0001 and *p* < 0.001, respectively.
8. Hazard ratio of survival for the treatment relative to the reference group.
9. Relative risk reduction; RRR = [1- Exp. (B) x 100)].
10. 95 % confidence interval for the hazard ratio.
