# Supplementary material for: Symbiont-Mediated Protection of Acromyrmex Leaf-Cutter Ants from the Entomopathogenic Fungus Metarhizium anisopliae
Source: mBio. 2021 Dec 21;12(6):e01885-21. doi: 10.1128/mBio.01885-21 (PMC8689564; doi:10.1128/mBio.01885-21)
Supplement: FIG S2 [file mbio.01885-21-sf002.docx]

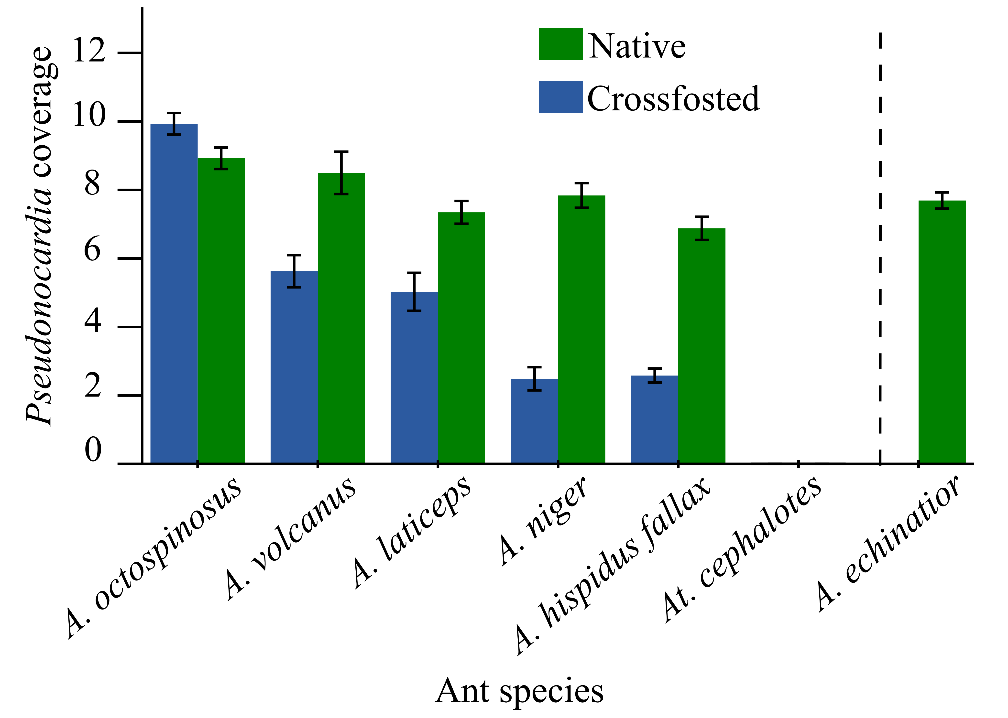


Figure S2. Graph comparing the difference in *Pseudonocardia* coverage (mean ± SE) on *Acromyrmex echinatior* workers carrying *Pseudonocardia* ectosymbiont from different leaf-cutter ant species (cross-fostered) and when these *Acromyrmex* species are reared by their conspecific nestmates (native colony). The bar within the dashed lines represents *A. echinatior* ants raised only by their own colony and *At. cephalotes*, which naturally does not carry *Pseudonocardia*, was used as negative control of *Pseudonocardia* acquisition.
