# Supplementary material for: Symbiont-Mediated Protection of Acromyrmex Leaf-Cutter Ants from the Entomopathogenic Fungus Metarhizium anisopliae
Source: mBio. 2021 Dec 21;12(6):e01885-21. doi: 10.1128/mBio.01885-21 (PMC8689564; doi:10.1128/mBio.01885-21)
Supplement: FIG S3 [file mbio.01885-21-sf003.docx]

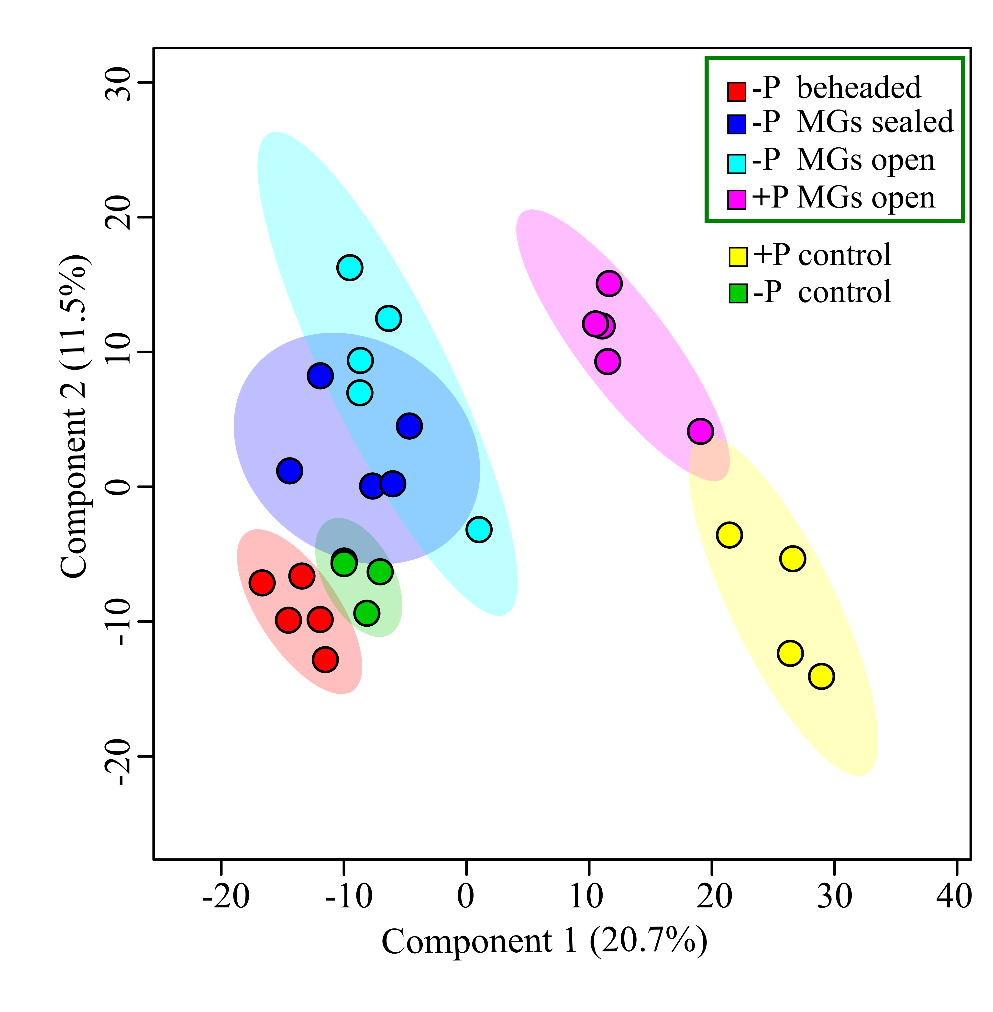


Figure S3. PLS-DA of the profile of metabolites associated with the ants’ exoskeleton treated with *Metarhizium* (inset green square) and control treatments. Dots on the graph represent a single ant per treatment clustered by a 95% confidence interval. Ants were reared in either *Pseudonocardia*-carrying condition (+P) with MG opened (pink) and MG opened control (yellow) or *Pseudonocardia*-free condition (-P) with MGs opened (light blue), MGs sealed (blue), beheaded (red), and MG opened control (green), and control denotes uninfected treatments.
