# Supplementary material for: Symbiont-Mediated Protection of Acromyrmex Leaf-Cutter Ants from the Entomopathogenic Fungus Metarhizium anisopliae
Source: mBio. 2021 Dec 21;12(6):e01885-21. doi: 10.1128/mBio.01885-21 (PMC8689564; doi:10.1128/mBio.01885-21)
Supplement: TEXT S1 [file mbio.01885-21-s0001.doc]

**ELECTRONIC SUPPLEMENTARY MATERIAL**

**Symbiont-mediated protection of *Acromyrmex* leaf-cutter ants from the entomopathogenic fungus *Metarhizium anisopliae***

Gaspar Bruner-Montero, Matthew Wood, Heidi A. Horn, Erin Gemperline, Lingjun Li & Cameron R. Currie

*DNA extraction, sequencing and phylogenetic analysis*

DNA of both ants and *Pseudonocardia* tissues were extracted using Epicenter Master Pure Complete DNA and RNA Purification Kit (Illumina, Madison, WI) with some modifications . Fresh tissues were homogenized using a Mini-beadbeater for 2.5 min in 2.0 mL screw-cap tubes with one 3 mm diameter sterile steel bead in 300 μl Tissue and Cell Lysis Solution. Subsequently, samples were centrifuged at 10,200 rpm for 10 min; the supernatant was collected and processed following the manufacturer’s protocols. Partial length sequence of the nuclear elongation factor gene (*EF-Tu*) was amplified using primers 52F and 920R that are specific for *Pseudonocardia* . Twenty-five μl total volume of PCR product was amplified using Gotaq Green Master Mix (Promega) at the following conditions: 94°C for 5 min, 40 cycles of 94°C for 30 s, 60°C for 45 s, 72°C for 2 min and a final extension at 72°C for 10 min. Amplicons were sequenced at the University of Wisconsin Biotechnology Center (http://biotech.wisc.edu).

All sequences were manually edited using Sequencher V 5.2 (Gene Code) and aligned with Clustal X and MUSCLE . Mega 6.0 was used to generate a Maximum likelihood phylogeny based on the Tamura-Nei method and bootstrapped 1000 times . Twenty-six sequences were added to the analysis from the GenBank database from previous studies for comparison.

*MALDI-Orbitrap imaging*

The ant thorax was removed from each ant and inlaid into custom glass slides. The slides were coated with MALDI matrix, 2, 5-dihydroxybenzoic acid, using an automatic matrix sprayer (HTX Imaging, Carrboro, NC) and positioned in the slide adapter MALDI plate. MALDI-MSI was performed with a high-resolution, accurate-mass MALDI-Orbitrap LTQ mass spectrometer with a spatial resolution of 75 µm in the mass range of *m/z* 100-1700. For more detail of the protocol see Gemerline *et. al.* (2017) .

**References**

[1] Hanshew, A.S., Mason, C.J., Raffa, K.F. & Currie, C.R. 2013 Minimization of chloroplast contamination in 16S rRNA gene pyrosequencing of insect herbivore bacterial communities. *J Microbiol Methods* **95**, 149-155. (doi:10.1016/j.mimet.2013.08.007).

[2] Poulsen, M., Cafaro, M., Boomsma, J.J. & Currie, C.R. 2005 Specificity of the mutualistic association between actinomycete bacteria and two sympatric species of *Acromyrmex* leaf-cutting ants. *Mol Ecol* **14**, 3597-3604. (doi:10.1111/j.1365-294X.2005.02695.x).

[3] Larkin, M.A., Blackshields, G., Brown, N.P., Chenna, R., McGettigan, P.A., McWilliam, H., Valentin, F., Wallace, I.M., Wilm, A., Lopez, R., et al. 2007 Clustal W and Clustal X version 2.0. *Bioinformatics* **23**, 2947-2948. (doi:10.1093/bioinformatics/btm404).

[4] Edgar, R.C. 2004 MUSCLE: multiple sequence alignment with high accuracy and high throughput. *Nucleic Acids Res* **32**, 1792-1797. (doi:10.1093/nar/gkh340).

[5] Tamura, K., Stecher, G., Peterson, D., Filipski, A. & Kumar, S. 2013 MEGA6: Molecular Evolutionary Genetics Analysis version 6.0. *Mol Biol Evol* **30**, 2725-2729. (doi:10.1093/molbev/mst197).

[6] Gemperline, E., Horn, H.A., DeLaney, K., Currie, C.R. & Li, L.J. 2017 Imaging with mass spectrometry of bacteria on the exoskeleton of fungus-growing ants. *Acs Chem Biol* **12**, 1980-1985. (doi:10.1021/acschembio.7b00038).

[7] Marsh, S.E., Poulsen, M., Pinto-Tomas, A. & Currie, C.R. 2014 Interaction between workers during a short time window is required for bacterial symbiont transmission in *Acromyrmex* leaf-cutting ants. *PLoS One* **9**, e103269. (doi:10.1371/journal.pone.0103269).

[8] Cafaro, M.J., Poulsen, M., Little, A.E., Price, S.L., Gerardo, N.M., Wong, B., Stuart, A.E., Larget, B., Abbot, P. & Currie, C.R. 2011 Specificity in the symbiotic association between fungus-growing ants and protective *Pseudonocardia* bacteria. *Proc Biol Sci* **278**, 1814-1822. (doi:10.1098/rspb.2010.2118).
